# Affinity maturation of TCR-like antibodies using phage display guided by structural modeling

**DOI:** 10.1093/protein/gzac005

**Published:** 2022-02-17

**Authors:** Rahel Frick, Lene S Høydahl, Ina Hodnebrug, Erik S Vik, Bjørn Dalhus, Ludvig M Sollid, Jeffrey J Gray, Inger Sandlie, Geir Åge Løset

**Affiliations:** Centre for Immune Regulation and Department of Immunology, University of Oslo and Oslo University Hospital, Sognsvannsveien 20, 0372 Oslo, Norway; Centre for Immune Regulation and Department of Biosciences, University of Oslo, Blindernveien 31, 0371 Oslo, Norway; Department of Chemical and Biomolecular Engineering, Johns Hopkins University, 3400 N. Charles Street, Baltimore, MD 21218, USA; Centre for Immune Regulation and Department of Immunology, University of Oslo and Oslo University Hospital, Sognsvannsveien 20, 0372 Oslo, Norway; Centre for Immune Regulation and Department of Biosciences, University of Oslo, Blindernveien 31, 0371 Oslo, Norway; KG Jebsen Coeliac Disease Research Centre, University of Oslo, Sognsvannsveien 20, 0372 Oslo, Norway; Centre for Immune Regulation and Department of Immunology, University of Oslo and Oslo University Hospital, Sognsvannsveien 20, 0372 Oslo, Norway; Centre for Immune Regulation and Department of Biosciences, University of Oslo, Blindernveien 31, 0371 Oslo, Norway; Nextera AS, Gaustadalléen 21, 0349 Oslo, Norway; Department for Medical Biochemistry, Institute for Clinical Medicine, University of Oslo, Sognsvannsveien 20, 0372 Oslo, Norway; Department for Microbiology, Clinic for Laboratory Medicine, Oslo University Hospital, Sognsvannsveien 20, 0372 Oslo, Norway; Centre for Immune Regulation and Department of Immunology, University of Oslo and Oslo University Hospital, Sognsvannsveien 20, 0372 Oslo, Norway; KG Jebsen Coeliac Disease Research Centre, University of Oslo, Sognsvannsveien 20, 0372 Oslo, Norway; Program in Molecular Biophysics, Johns Hopkins University, 3400 N. Charles Street, Baltimore, MD 21218, USA; Department of Chemical and Biomolecular Engineering and Institute of NanoBioTechnology, Johns Hopkins University, 3400 N. Charles Street, Baltimore, MD 21218, USA; Sidney Kimmel Comprehensive Cancer Center, Johns Hopkins School of Medicine, 733 N Broadway, Baltimore, MD 21205, USA; Centre for Immune Regulation and Department of Immunology, University of Oslo and Oslo University Hospital, Sognsvannsveien 20, 0372 Oslo, Norway; Centre for Immune Regulation and Department of Biosciences, University of Oslo, Blindernveien 31, 0371 Oslo, Norway; Centre for Immune Regulation and Department of Immunology, University of Oslo and Oslo University Hospital, Sognsvannsveien 20, 0372 Oslo, Norway; Centre for Immune Regulation and Department of Biosciences, University of Oslo, Blindernveien 31, 0371 Oslo, Norway; Nextera AS, Gaustadalléen 21, 0349 Oslo, Norway

**Keywords:** antibody, phage display, celiac disease

## Abstract

TCR-like antibodies represent a unique type of engineered antibodies with specificity toward pHLA, a ligand normally restricted to the sensitive recognition by T cells. Here, we report a phage display-based sequential development path of such antibodies. The strategy goes from initial lead identification through *in silico* informed CDR engineering in combination with framework engineering for affinity and thermostability optimization, respectively. The strategy allowed the identification of HLA-DQ2.5 gluten peptide-specific TCR-like antibodies with low picomolar affinity. Our method outlines an efficient and general method for development of this promising class of antibodies, which should facilitate their utility including translation to human therapy.

## Introduction

Antibodies with high specificity, stability and affinity are important as therapeutic agents against a range of diseases and as research tools. However, generating human antibodies with these properties against defined targets can still be difficult. Such antibodies can be isolated from immune receptor transgenic animals or from human individuals with a relevant immune profile, for example after viral infections. These antibodies undergo affinity maturation *in vivo* and thus tend to have high affinity. However, *in vivo* generation offers limited control over epitope targeting and fine specificity and may not be effective at identification of a desired antibody lead with strict requirements to binding properties. If antibodies are isolated from naïve or synthetic libraries by use of display technologies, such as phage display or yeast display, the control of fine specificity as well as developability may be improved, but the primary leads tend to have suboptimal target affinity. Therefore, initial candidate antibodies are routinely subjected to laborious rounds of engineering to enhance their affinity, and occasionally, to refine their specificity. Most often, this is accomplished by introduction of random mutations into an antibody fragment variable (Fv) region and/or targeted mutagenesis in the Complementarity Determining Region (CDR) followed by a secondary selection (Kiguchi *et al.,* 2021; Lee *et al.,* 2021).

If a high-resolution structure of a lead antibody in complex with its antigen is available, one may choose mutations rationally and focus them to sites that are most likely to affect affinity (Nelson *et al.,* 2018), but the generation of such structures may be time-consuming, expensive or impossible. Moreover, the static nature of crystal structures fails to embrace the inherent dynamic features of the antibody (Fernández-Quintero *et al.,* 2020). There are also algorithms for computational affinity maturation of antibodies that directly predict enhancing mutations (Li *et al.,* 2014; Adolf-Bryfogle *et al.,* 2018; Chowdhury *et al.,* 2018; Kuroda and Tsumoto, 2018; Warszawski *et al.,* 2019). Typically, these methods require screening of a relatively large number of mutants and rely heavily on a high-resolution crystal structure to accurately predict mutations. The dependence on crystal structures is likely to be partly relieved by rapidly improving machine learning (ML) methods for protein structure prediction and design for antibodies and other classes of proteins (Anishchenko *et al.,* 2020; Abanades *et al.,* 2021; Baek *et al.,* 2021; Jumper *et al.,* 2021; Ruffolo *et al.,* 2022; Ruffolo and Gray, 2022).

In this study, we applied a combination of computational and experimental tools to achieve affinity maturation of the previously reported human T-cell receptor (TCR)-like antibody clone 107 (Høydahl *et al.,* 2019a). It binds a human leukocyte antigen (HLA) peptide complex (pHLA), specifically HLA-DQ2.5, in complex with the immunodominant celiac disease epitope DQ2.5-glia-α1a. pHLA represents a class of antigens that continues to be a challenging target in antibody discovery campaigns, both due to experimental hurdles as well as difficulty in acquiring experimental structural information (Holland *et al.,* 2020). Here, we show how to use computational models to inform rational CDR library design in the absence of a crystal structure of the antibody:antigen complex, or even of the antibody alone.

Directed by analysis of computational models of the antibody:antigen complex, we generated two focused antibody libraries with mutations in the CDRs (targeting CDR H1 or H3), as well as a generic library containing random mutations introduced by error-prone PCR. After selection, clones with desired properties were isolated from both types of libraries. The increase in affinity was considerably larger in the CDR-targeted structure-guided approach, while the improvement in thermostability was superior in the random mutagenesis approach. From these primary clones, we also generated a secondary variant termed 4.7Cplus, which combined the mutations of the best target binder with those of the most stable variant, and 4.7Cplus outperformed all other clones in binding its target with an affinity of about 20 pM (Kd), which is a 3500-fold increase in monomeric affinity compared with the parent clone 107. This is one of the highest affinity fully human TCR-like antibodies reported to date (Liu *et al.,* 2017; Høydahl *et al.,* 2019b). In summary, we here outline a general strategy that can easily be adopted by most antibody engineering laboratories to facilitate improved discovery and development of TCR-like antibodies.

## Results and Discussion

### Structural model of the antibody 107 and library design

**Fig. 1:**
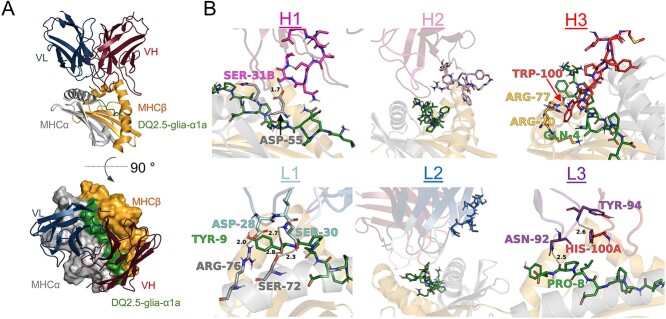
Fv-pHLA model and library design. (A) Low-scoring models suggest a diagonal binding mode across the antigen-binding groove. (B) All six CDR loops were analyzed regarding their putative contribution to both peptide and HLA binding and their potential for improvement. The CDR loops as well as potentially crucial contacts in the pHLA are shown in stick representation and interacting residues are annotated.

We have previously described the isolation and use of the TCR-like antibody clone 107 specific for HLA-DQ2.5:DQ2.5-glia-α1a (Høydahl *et al.,* 2019a). We had generated structural models of the antibody Fv domain using RosettaAntibody and predicted its interaction with the corresponding HLA-DQ2.5 complexes using SnugDock (Høydahl *et al.,* 2019a) (Fig. S1, Supplementary data are available at *PEDS* online). Here, we sought to improve its target affinity beyond the current dissociation constant (Kd) of about 70 nM. In our structural model of the antibody:pHLA complex, clone 107 was predicted to bind pHLA in a diagonal TCR-like manner, with the footprint focused on the C-terminal part of the peptide (Fig. 1A). The light chain loops L1 and L3 were predicted to have favorable polar interactions with both the peptide and the HLA (D28, S30, N92 and Y94) (Fig. 1B). The CDR L2 loop was oriented away from the antigen in our model. The heavy chain on the other hand was predicted to be suboptimal for binding (Fig. 1B). The H1 loop was predicted to form one hydrogen bond with the HLA α-chain using the side chain of S31B, but the remaining side chains were placed too far away from the pHLA to directly interact. The CDR H2 loop was oriented away from the peptide similar to the light chain CDR2 loop. The CDR H3 loop, which is often the most important determinant for specificity and affinity and contributes a large part to the buried surface area at the interface, was predicted to be in direct contact with the peptide and the HLA. A central Trp (W100) stood out for its placement in a pocket surrounded by side chains of the peptide (Q4) and the HLA β-chain (R70 and R77), but we observed few other direct contacts. We hypothesized that variation in the CDR H1 and H3 loops would either improve existing interactions or introduce new ones to increase target affinity. We therefore generated two focused libraries using degenerate oligonucleotides (NNK): one library with randomized sequence and increased length (plus 1–2 amino acids) of the CDR H1 loop and a second library with randomized sequence of the CDR H3 loop (Table S1, Supplementary data are available at *PEDS* online). Since the model of 107 positioned a tryptophan (W100) in a pocket between the peptide and the HLA α-chain suggesting that this residue may be important for binding, we retained this residue in 50% of the H3 library clones. We achieved 1–5 × 10^9^ primary transformants into *Escherichia coli* SS320 host cells for both the CDR H1 and H3 libraries. Additionally, as paratope-distal residues may improve biophysical properties and thus affect affinity (Low *et al.,* 1996; Hanes *et al.,* 2000; Raghunathan *et al.,* 2012; Koenig *et al.,* 2017), we generated a random library with mutations across the entire single-chain (sc)Fv sequence by error-prone PCR using dNTP analogues, achieving an average amino acid mutagenesis load of 4% and 5 × 10^7^ primary transformants.

### Selection of high-affinity gluten pHLA-specific antibodies

**Fig. 2:**
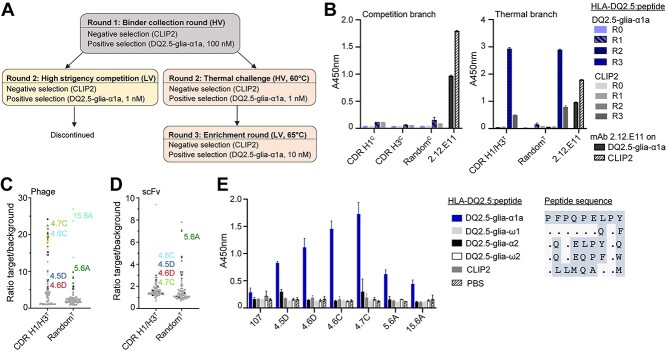
Selection and screening of antibody libraries. (A) Overview of the selection strategy. Libraries were packaged with either M13K07 or DeltaPhage helper phages to achieve LV and HV display, respectively. After R1, the libraries were selected in a competition branch and a thermostability branch in parallel. (B) Polyclonal phage ELISA to assess enrichment of binders against HLA-DQ2.5:DQ2.5-glia-α1a in the phage outputs after R1-R3. HLA-DQ2.5:CLIP2, which was used for negative selection, was used to monitor HLA-DQ2.5 binding irrespective of peptide. (C and D) The selection outputs after three rounds of panning were screened in HV format (C) and scFv format (D) to assess binding to target pHLA complexes and HLA-DQ2.5:CLIP2 (background) in ELISA and signal/noise ratios were calculated. Each dot represents one clone. Gray dots denote unknown sequences, black dots denote unique amino acid sequences and colors represent enriched sequences. Both libraries were selected in the thermostability branch. (E) Binding of purified Fab fragments (5 μg/mL) to different HLA-DQ2.5:peptide complexes was assessed by ELISA. Error bars illustrate mean ± SD of duplicates (*n* = 2). Alignment of 9mer core peptide sequences is shown on the right.

For the selection of second-generation antibodies, we used a phage display selection strategy that we have described previously (Frick *et al.,* 2021) (Fig. 2A). Briefly, all primary libraries were packaged at high valence (HV) displayed on phage coat protein pIX (Nilssen *et al.,* 2012) and selected in an initial low-stringent round (R1). For the subsequent rounds, the libraries were split into a thermostability branch and a competition branch and subjected to a selection campaign based on the hammer-hug selection protocol with a stringent R2 followed by a non-stringent R3 (Fennell *et al.,* 2013). In R2 of the competition branch, high stringency was induced by displaying scFvs at low valence (LV) and forcing competition of library members for low target amounts in the presence of the 107 parent clone in IgG format. In the thermal branch, scFvs were displayed at HV and heat challenged at a temperature inducing unfolding of the parent clone prior to the selection to aggregate and remove unstable library members (Fig. S2A, Supplementary data are available at *PEDS* online) (Jespers *et al.,* 2004; Gunnarsen *et al.,* 2013). In the low-stringent R3, we increased antigen concentration to recover and amplify selected binders. For the thermal branch, libraries went through a second heat challenge at LV display to select clones with high monomeric affinities. All libraries selected in the competition branch had close to no selection output after R2 and were therefore discontinued. This may be due to an overly abundant parent clone with relatively high affinity making the competition too stringent or low expression of the variant clones on phage in the LV display in R2. In a similar selection with a lower affinity parent clone and library members with higher affinity and expression levels (Fig. S2B, Supplementary data are available at *PEDS* online), we did obtain output from the competition branch (Frick *et al.,* 2021). In contrast, we observed output from both the random and the CDR-targeted libraries in the thermostability branch. To determine antigen reactivity of the output, we performed a polyclonal phage ELISA (Fig. 2B). Indeed, both R3 outputs showed antigen binding. Next, we randomly picked single clones from the R3 output and screened by ELISA for antigen binding of scFvs displayed on phage (Fig. 2C) and as soluble scFv (Fig. 2D). Several clones from the random library bound modestly as scFv and displayed on phage, while the CDR-targeted library clones only gave high signals when displayed on phage. In a previous study, we noted that the leader peptide-independent pIX phage system favors high stability and ongoing work suggests that this may translate to the dichotomy of high display levels on phage and low soluble expression in standard leader peptide-dependent bacterial expression (Høydahl *et al.,* 2016). We observed 64 clones with target/background binding >3 from the CDR-targeted library and 19 from the random library. Sequencing of 50 offspring from the CDR-targeted libraries revealed 35 unique DNA sequences and 17 unique amino acid sequences. All of these originated from the CDR H3 library and all possessed the central W100 residue, confirming its importance for binding. We further sequenced 24 clones from the random library and identified a sequence denoted 5.6A in 15 out 24 clones.

Based on single-clone target binding in screening and analysis of enriched sequence features, we chose 6 unique clones for Fab expression in HEK293E cells, all of which expressed well in this eukaryotic system. Analysis of peptide-specificity in ELISA, using a panel of HLA-DQ2.5:peptide complexes showed that all bound HLA-DQ2.5 with DQ2.5-glia-α1a specifically (Fig. 2E). None of the clones cross-reacted to related gliadin epitopes and of particular importance, the highly similar DQ2.5-glia-ω1 complex, which differs at only two positions of the peptide (p7 and p9) (Fig. 2E). We assessed the structural model of the antibody:pHLA complex but were unable to identify direct polar interactions that would explain the observed specificity for positions p7 and p9. The predicted antibody footprint was shifted slightly toward the C-terminus of the peptide compared with T cell receptors with the same specificity (Petersen *et al.,* 2014), and the model predicted the placement of several residues in the antibody light and heavy chains within 4 Å of the relevant peptide positions (Fig. S3A, Supplementary data are available at *PEDS* online), suggesting that hydrophobic interactions or indirect polar interactions may confer specificity. Such subtle effects also appear to underly the fine-tuned ability to discriminate between the DQ2.5-glia-α1a and DQ2.5-glia-ω1 epitopes seen with the majority of T-cell clones isolated from celiac disease patient (Dahal-Koirala *et al.,* 2019).

### Biophysical characterization and construction of the combination variant 4.7Cplus

**Fig. 3:**
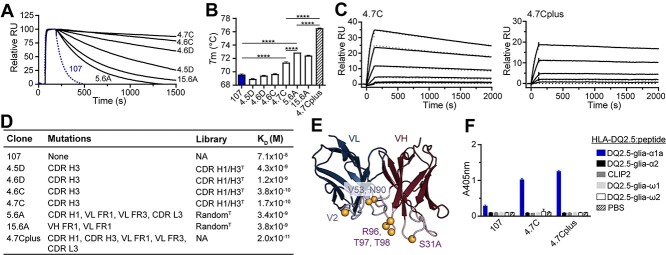
Biophysical characterization of leads. (A) Binding of Fab fragments to HLA-DQ2.5:DQ2.5-glia-α1a by SPR. Fabs were ranked based on off-rates. The parent clone 107 is shown in blue (*n* = 2). (B) Melting temperatures (Tm) of the parent clone 107 (blue) and the affinity matured Fab fragments. Error bars illustrate mean ± SD of 3–7 replicates. Statistical analysis was performed by unpaired two-tailed *t*-test, ^****^P < 0.0001. (C) Representative sensorgrams of 4.7C and combination variant 4.7Cplus (*n* ≥ 2). Data were fitted to a 1:1 Langmuir binding model (dotted gray lines). (D) Table detailing position of mutations, library origin and Kd value of the individual clones. FR, framework; NA, not applicable. (E) The locations of the mutations present in the combination mutant 4.7Cplus are illustrated as spheres. VH contains the following mutations: CDR H1 Asn 31A Ser and CDR H3 Ser 96 Arg, Ser 97 Thr and Ser 98 Thr. VL contains the following mutations: FR1 Ile 2 Val, FR3 Ile 53 Val and CDR L3 Asp 90 Asn. The Fv model is based on mAb 107. (F) The candidate antibodies were reformatted to full-length hIgG1 (0.5 μg/mL) and binding to a panel of related soluble peptide:HLA-DQ2.5:gliadin complexes or controls was analysed by ELISA. Error bars illustrate mean ± SD of duplicates (*n* = 3).

To determine if the candidate clones had higher target affinity than the parent clone, we performed binding analysis with monomeric Fabs using surface plasmon resonance (SPR) and ranked the clones based on their off-rates (Fig. 3A). To alleviate re-binding events, we used a low pHLA coupling density and a high-flow-rate during SPR measurements. Strongly reduced off-rates were observed for all clones tested. The two clones from the random mutagenesis library, 5.6A and 15.6A, had less pronounced affinity improvements than the four CDR H3 mutants selected from the library using structural models, underscoring the additional benefit of the targeted approach. Based on the results, we chose 4.7C as lead for binding to HLA-DQ2.5:DQ2.5-glia-α1a.

We next assessed the thermostability of all Fab fragments by determining their melting temperatures using nanoDSF (Fig. 3B), and 3 of the 6 clones improved compared with the parent clone 107. The lead clone 4.7C had the highest thermostability out of the clones isolated from targeted library with an increase in Tm of about 1.9°C (Fig. 3B). In line with the rational for generating the random mutation libraries, the clones 5.6A and 15.6A had the largest improvement in thermostability (3.3 and 2.5°C, respectively). This increased stability and corresponding improvement in functional display also explains why screening of the random library in scFv format gave stronger signals than screening the CDR-targeted library. Since additive effects may be gained by combining favorable mutations separately affecting stability and target binding, we constructed a combination variant termed 4.7Cplus, combining the CDR H3 sequence of 4.7C and with the sequence of clone 5.6A. Indeed, the 4.7Cplus variant displayed a synergistic effect in stability translating to a 6.9°C (±0.1°C) increase in *T_m_* from the 107 mother clone (Fig. 3B).

**Fig. 4:**
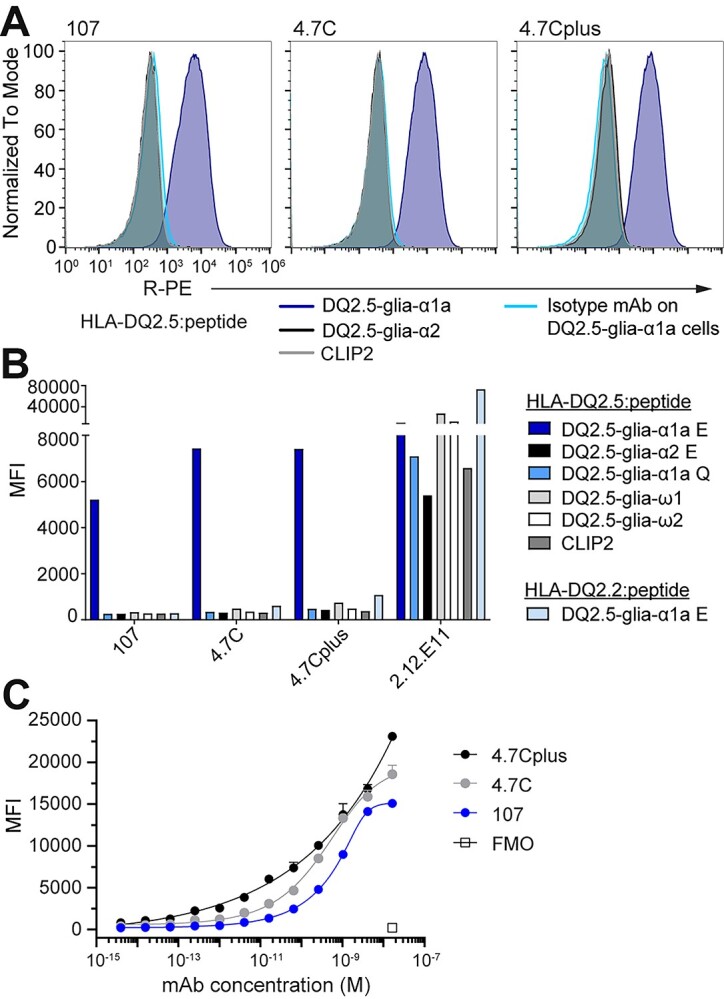
The lead mAbs bind pHLA specifically on a cell surface. Murine A20 B cells engineered to express HLA-DQ2.5 with covalently linked peptide were stained with 107, 4.7C or 4.7Cplus hIgG1 or a hIgG1 isotype control mAb (5 μg/mL). (A) Histograms show mAb binding to A20 HLA-DQ2.5 with DQ2.5-glia-α1a (TCR-like mAbs in blue, isotype mAb in turquoise), DQ2.5-glia-α2 (black) or CLIP2 (gray) (*n* = 2). (B) MFI of mAb binding to the complete panel of A20 B cells expressing different pHLAs; 2.12.E11 mIgG1 was included as control (*n* = 2). (C) Binding slopes of mAbs 107, 4.7C and 4.7Cplus binding to A20 B cells expressing HLA-DQ2.5 with DQ2.5-glia-α1a. mAbs were titrated from 16.5 nM (4-fold dilution) and binding was analyzed by flow cytometry and visualized as MFI values. Error bars illustrate mean ± SD of duplicates (*n* = 2–4).

In concordance with the improved (lower) off-rates, all candidates had a strong improvement in affinity, with 4.7C having a dissociation constant Kd of about 170 ± 40 pM (Fig. 3C and D) representing a 400-fold improvement over the parent clone 107 which has an Kd value of about 70 nM. The combination variant 4.7Cplus, which combines mutations in several CDR and framework regions, had a further improved affinity with Kd estimated to about 20 pM in SPR (Fig. 3C–E, Table S3, Supplementary data are available at *PEDS* online). The second-generation antibodies were then expressed as full-length hIgG1 and tested for specific binding in ELISA (Fig. 3F). In agreement with the observations for the Fab fragments, both 4.7C and 4.7Cplus bound exclusively to the target complex HLA-DQ2.5:DQ2.5-glia-α1a. Thus, the high affinity antibodies maintained the high specificity of the parent clone.

### Binding to cell-surface antigen

To further validate mAb specificity in a defined cellular context, we determined mAb binding using model A20 B cells transduced to ectopically express HLA-DQ2.5 with linked peptides to avoid variation in loading and decay effects (Fig. 4), and 107, 4.7C and 4.7Cplus all bound to cells presenting DQ2.5-glia-α1a, but not to the control DQ2.5-glia-α2 and CLIP2 versions (Fig. 4A). Notably, the high-affinity variants, 4.7C and 4.7Cplus, bound with a higher median fluorescent intensity (MFI) than mAb 107 (Fig. 4B). In line with earlier results, the mAbs did not bind to cells expressing a range of HLA-DQ2.5:peptide controls, including the DQ2.5-glia-ω1 peptide and a native, non-deamidated version of DQ2.5-glia-α1a (Q instead of E at peptide residue 6). Neither did the mAbs bind cells expressing HLA-DQ2.2 (DQA1^*^02:01/DQB1^*^02:01), a close homologue of HLA-DQ2.5 (DQA1^*^05:01/DQB1^*^02:01), in complex with the DQ2.5-glia-α1a peptide (Fig. 4B). We again turned to the structural model of 107 to see if it can explain the experimentally observed specificity. Of the 10 membrane-distal polymorphic residues in these two α-chains (DQA) only the S72I polymorphism is surface exposed and located at or near the TCR binding site (Fig. S3B, Supplementary data are available at *PEDS* online). The predicted C-terminal footprint of 107 puts position S72 within reach of the antibodies, and the model predicts a hydrogen bond between this position and S30 of the antibody light chain (Fig. S3C, Supplementary data are available at *PEDS* online). In effect, the mAbs have an exquisite HLA-specificity that even goes beyond that seen with some primary T cell isolates from CeD patients (Fallang *et al.,* 2009) which are typically cross-reactive to the HLA-DQ2.2 complex. In summary, the antibodies share major epitope and HLA specificity determinants with CeD patient derived TCRs including the DQ2.5-glia-α1a positions p6, p7 and p9, and uniquely sense S72 in the HLA-DQ2.5 α-chain. The high specificity of these antibodies both toward the target HLA molecule and the target peptide makes them promising candidates for research tools and therapeutics. Further adding to that promise is the fact that HLA-DQ2.5 stands out from other HLA class II molecules by presenting a very narrow peptide repertoire *in vivo*, partly due to their inefficient HLA-DM-mediated peptide exchange, leaving universal CLIP1/2 peptides the dominating peripheral epitopes (Fallang *et al.,* 2009). The narrow peptide repertoire, combined with the strict specificity for HLA-DQ2.5 and the target peptide, suggests that the risk of off-target reactivity is lower than for other TCR-like antibodies.

Finally, we tested binding of the antibodies in serial dilutions to the A20 cells expressing HLA-DQ2.5:DQ2.5-glia-α1a. All three mAbs bound in a concentration-dependent manner, and the two high affinity versions with higher sensitivity than the parent clone 107 (Fig. 4C). In this assay, we also observed a difference between the 4.7C and 4.7Cplus variants especially at lower mAb concentration pointing to the benefit of very high affinity. Despite the vastly improved monomeric affinity over the 107 parent clone, the added benefit of the two high affinity variants in detecting cell-surface antigen was relatively modest in the mid to high Ab concentration range using these engineered A20 cells. This is likely explained by 107 already having relatively high affinity and the supra-physiological density of ectopic antigen on the A20 cell surface leading to an avidity effect that masks the difference in monomeric affinity. In contrast, endogenous HLA class II is expressed at low density and only a fraction of these molecules will present the DQ2.5-glia-α1a peptide (Kowalewski *et al.,* 2015; Høydahl *et al.,* 2019a). Ongoing studies may clarify and broaden our understanding of specific *in situ* gliadin peptide presentation using these new high affinity reagents.

## Conclusions

We describe an approach for structure-guided affinity maturation of antibodies in absence of an experimental structure of the antibody or the complex. Our protocol relies on computational structural models combined with phage display selection and can be applied to a range of affinity maturation campaigns. Antibodies specific to pHLA are also called TCR-like antibodies and show promise in diagnosis and therapy of autoimmune diseases and cancer (Høydahl *et al.,* 2019b) but are difficult to engineer to achieve the necessary affinity and specificity, and few examples exist that target HLA class II (Xu *et al.,* 2019; Holland *et al.,* 2020). Clone 107 in this study was initially isolated from a fully human naïve scFv library and targets a prominent pHLA class II important in celiac disease with intermediate affinity (Høydahl *et al.,* 2019a). We thus wanted to see if target binding could be improved by affinity maturation but lacked structural data for directed mutagenesis or library design. However, experimental data that show specificity to the peptide effectively limit the search area for docking from a global search to a local search making this a well-suited problem for structural modeling. This affinity maturation campaign showcases that even in the absence of experimental structural information, a structure-guided approach can produce TCR-like antibodies with substantially increased affinity compared with the parent clone and to clones isolated from unbiased random libraries. While the CDR H3 loop is a common target for affinity and specificity engineering studies, the structural models additionally suggested the CDR H1 loop as a possible target and pointed out a crucial Trp residue at the tip of the H3 loop, which was maintained in all sequenced clones from the selection output which confirms its importance for binding. The antibody clones reported here are part of a panel of celiac disease-specific antibodies involved in further studies which should greatly benefit from the extraordinarily high sensitivity in pHLA binding achieved.

The structural model of the antibody:antigen complex was generated using RosettaAntibody and Rosetta SnugDock (Weitzner *et al.,* 2017; Høydahl *et al.,* 2019a) which are leading physics-based methods for antibody structure prediction and docking, on par with comparable methods at the time of this study (Almagro *et al.,* 2014; Guest *et al.,* 2020). Since the inception of this study, ML-based methods such as AlphaFold2 and RoseTTAFold have revolutionized the field of protein structure prediction and design (Baek *et al.,* 2021; Jumper *et al.,* 2021) and new ML-based methods for antibody structure prediction outperform traditional ones (Ruffolo *et al.,* 2020, 2022; Abanades *et al.,* 2021; Ruffolo and Gray, 2022). ML methods have also been applied to the protein docking problem (Evans *et al.,* 2021) but have so far mostly failed at predicting antibody:antigen complexes (Yin *et al.,* 2021), suggesting that models continue to be uncertain and experimental methods for affinity maturation will still play a role. In summary, the ongoing development and rapid improvement of methods can be harnessed in similar engineering projects building on higher confidence models and thus likely further strengthening the usefulness of the engineering scheme outlined here.

## Material and Methods

### Library construction

The CDR-targeted libraries were constructed using the sequence of the scFv 107 parent clone with AgeI restriction enzyme sites inserted into the target site as template (for later removal of template background) using custom-designed oligonucleotides containing NNK-codons as described before (Tonikian *et al.,* 2007; Frick *et al.,* 2021). The random library based on parent clone 107 was generated using the JBS dNTP-Mutagenesis Kit (Jena Biosciences, Jena, Germany), followed by template DNA removal by DpnI digestion. The final libraries were transformed into *E. coli* SS320 (Lucigen, Middleton, WI, USA) and packaged for HV display using Deltaphage. Library construction and packaging is detailed in Supplementary Materials and Methods.

### Phage display selection and screening of binders

Selection of phage libraries (strategy outlined in Fig. 2A) and screening of single clones are detailed in Supplementary Materials and Methods. Briefly, phage libraries were selected on recombinant, biotinylated pHLA in solution. Following negative selection using a control pHLA complex, antigen-binding clones were captures onto streptavidin-coated magnetic beads (Dynabeads MyOne Streptavidin T1, Invitrogen, Waltham, MA, USA). *Escherichia coli* SS320 were infected with eluted phages for packaging of the selection output. After R3, single clones were screened for antigen-binding in both scFv and phage format.

### Recombinant protein production, biophysical properties and analysis of binding specificity

The Supplementary Materials and Methods details the assessment of biophysical properties and binding specificity of the candidate clones by ELISA, SPR, nanoDSF and by flow cytometry using various antibody formats (scFv, Fab or full-length IgG).

## Authors’ Contributions

R.F., L.S.H., I.H., E.S.V. and B.D. designed and performed research and analyzed data. J.J.G., L.M.S., I.S. and G.Å.L. designed research, analyzed data and supervised the study. R.F., L.S.H. and G.Å.L. wrote the manuscript. All authors critically reviewed the manuscript.

## Supplementary Material

PEDS_a1a_engineering_SI_clean_1_pzac005

## Notes

Edited by: Feng Ni.
