# Supplementary material for: Affinity maturation of TCR-like antibodies using phage display guided by structural modeling
Source: Protein Eng Des Sel. 2022 Feb 17;35:gzac005. doi: 10.1093/protein/gzac005 (PMC9536190; doi:10.1093/protein/gzac005)
Supplement: PEDS_a1a_engineering_SI_clean_1_pzac005 [file peds_a1a_engineering_si_clean_1_pzac005.docx]

# Supplementary Materials and Methods

## Antibody modeling and docking to pHLA

A structural model of the 107 Fv fragment was generated as described *(1)* and reported *(2)*. Briefly, homology models were generated using RosettaAntibody and docked to HLA-DQ2.5:DQ2.5-glia-α1a (1S9V) *(3)* using Rosetta SnugDock *(4, 5)* with the T-cell receptor structure in a ternary TCRpHLA complex (4OZI) *(6)* as a template for an initial orientation. Models were selected for interpretation and visualization based on low Rosetta interface score (I_sc) and similarity to other low scoring models.

## Generation of targeted and randomized scFv phage libraries

The **CDR-targeted libraries** were based on scFv sequence of the parent clone 107 in pGALD9ΔLFN *(7)* and were generated using a modified version (reported in *(8)*) of a previously described protocol *(9)*. Briefly, to allow for removal of template background, we inserted AgeI restriction enzyme sites into the target sequences of the parent clone using mutagenic oligonucleotides and standard PCR techniques. Phage carrying this template was then packaged from *E. coli* XL1-Blue by superinfection with M13K07 (MOI 10), followed by PEG/NaCl precipitation (20 % w/v PEG 8,000, 2.5 M NaCl) and extraction of single-stranded (ss) DNA using a QIAprep Spin M13 kit. Mutagenic oligonucleotides containing 6-7 degenerate NNK codons (N: A/C/G/T, K: T/G) were designed to target selected positions of the CDR H1 or H3 loops enabling sequence randomization and length variation of the loops. 5’-phosphorylated oligonucleotides were annealed to the ssDNA, followed by synthesis of heteroduplex DNA using T7 DNA polymerase and ligation using T4 DNA ligase. Electrocompetent *E. coli* AVB100FmkII *(10)* was transformed with agarose gel purified covalently closed circular double-stranded DNA using a BTX ECM 600. Transformation mixtures were rescued with SOC medium 30 min/37 °C/200 rpm, centrifuged and plated onto LB-TAG agar Bioassay dishes (245 mm x 245 mm, Nunc) and grown at 30 °C/16 h. Additionally, primary transformations were spot titrated onto nitrocellulose membranes to determine library sizes. DNA was isolated from material of scraped plates and digested with AgeI to remove template. Undigested library DNA was purified from agarose gels, transformed into electrocompetent *E. coli* SS320 (Lucigen) and plated onto LB-TAG agar Bioassay dishes before phage packaging as described *(11)* using DeltaPhage *(12)* for HV display on coat protein pIX. Phage samples were 2x PEG/NaCl precipitated before selection.

The **random library** based on mother clone 107 was generated using the JBS dNTP-Mutagenesis Kit (Jena Bioscience) according to manufacturer instructions. Template DNA was removed by DpnI digest. Sequence analysis revealed that the amino acid mutation frequency was 4% and mutations were distributed uniformly along the scFv cassette. The total diversity of this library was estimated to 5x10^7^ by spot titration onto nitrocellulose membranes. Mutated scFv were subcloned into the pGALD9ΔLFN vector. DNA was purified by ethanol precipitation (Pellet Paint TM, Novagen) and transformed into *E. coli* SS320 (Lucigen) and packaged as described above.

## Phage display selection for affinity maturation and screening of selection outputs

The selection was performed essentially as described *(8)*. In brief, pre-blocked (4% PBSMT) phage libraries (R1 input of 3.0x10^11^ cfu^ampR^) were selected on recombinant, biotinylated pHLA in solution (4% PBMST), followed by capture onto pre-blocked (4% PBMST) streptavidin-coated magnetic beads (Dynabeads MyOne Streptavidin T1, Invitrogen). All positive selections in R1-R3 were preceded by a negative selection on HLA-DQ2.5:CLIP2. A pHLA target antigen concentration of 100 nM was used in R1, followed by reduction to 1 nM and 10 nM in R2 and R3, respectively as detailed in Figure 2A. Bead-captured pHLA:phage were washed 5+5 (PBST and PBS) in R1, and increased to 10+10 in R2 and R3. Before R2 and R3 of the thermal branch, non-blocked phage samples were heated for 15 min to 60 °C and 65 °C, respectively, before they were placed on ice and centrifuged at 17,000xg/5 min. During positive selection in the competition branch, bead-captured pHLA:phage particles were incubated in the presence of 50 nM of the hIgG1 107 parent clone. Phages were eluted using 0.5% trypsin (Gibco) for 15 min at 37°C and subsequently half the eluate was used to infect fresh log-growth *E. coli* S320. Cells were plated on 20x20 cm LA-TAG Q-trays grown ON/30°C, scraped and new phage packaged as specified for library generation. For low valence (LV) and high valence (HV) display, *E. coli* were superinfected with M13K07 or DeltaPhage, respectively.

## Screening of selection output

Random, single clones of the R3 output were picked and expressed in either scFv or phage format to screen for antigen-binding as described. Screening of the selection output in soluble scFv format was performed as described below and detailed in *(8)*. For screening in phage format, single clones were expressed and packaged in 96-deep well plates as described *(8)*. Briefly, clones were inoculated into YT-AG and cultured ON/37 °C/600 rpm. 10 µl were transferred to new plates containing fresh medium and grown for 3 h before superinfection with 10^9^ cfu DeltaPhage per well. Plates were incubated at 37 °C/30 min with gentle agitation, and further 30 min with vigorous shaking before cells were pelleted and resuspended in 50 µl 2x YT-AK and phage were packaged ON/30 °C. 100 µL cleared supernatants were used for screening in ELISA.

## Recombinant pHLA expression and purification

Recombinant, soluble pHLA were produced in insect cells and affinity purified using the monoclonal antibody 2.12.E11 as previously described *(13)*. Complexes containing the following peptides (9mer core is underlined) covalently linked to the HLA β-chain (linker GAGSLVPRGSGGGGS) were used; DQ2.5-glia-α1a (QLQPFPQPELPY), DQ2.5-glia-α2 (PQPELPYPQPE), CLIP2 (MATPLLMQALPMGAL), DQ2.5-glia-ω1 (QQPFPQPEQPFP), DQ2.5-glia-ω2 (FPQPEQPFPWQP), DQ2.5-glia-γ2 (QGIIQPEQPAQL), and DQ2.5-hor-3 (EQPIPEQPQPYP). The pHLA molecules were biotinylated in a site-specific manner using BirA. Prior to SPR, monomeric pHLA were isolated by size exclusion chromatography using a Superdex 200.

## Recombinant antibody expression and purification

For *E. coli* expression, scFv cassettes were either individually subcloned or batch-cloned as NcoI/NotI fragments from phagemids into the pFKPEN vector containing a His-tag as well as a Myc-tag *(14)*. Following transformation of electrocompetent *E. coli* XL1-Blue, protein was expressed in 96-deep well plates using 400 µl culture medium for screening of clones or in 1 L cultures for large-scale expression essentially as described *(14)*. Of note, 2x YT was used as culture medium and 0.1 M IPTG was used for induction of protein expression. For 96-deep well cultures 50 µL of the initial overnight culture was used to re-inoculate new cultures for expression. Periplasmic fractions were filtered, diluted by adding 100 mL PBS supplemented with 150 mM NaCl and 0.05% sodium azide, and adjusted to pH 7.4 before purification. For eukaryotic mAb expression, variable regions were cloned into pLNOH2 oriP and pLNOκ oriP expression vectors encoding constant human γ1 and constant human κ domains (cloned as BsmI/BsiWI fragments, GenScript) *(15)* or constant mouse IgG2b and constant mouse κ domains (cloned as BsmI/BamHI fragments, GenScript) *(2)* for generation of hIgG1 and mIgG2b mAbs, respectively. For expression of Fab fragments, the VH-encoding BsmI/BsiWI fragments used to generate the hIgG1 mAb was cloned into a vector for Fab expression *(2)*. mAbs and Fab fragments were expressed in HEK293E cells (ATCC) as described *(15)*. Cells were cultured in RPMI supplemented with 10 % fetal calf serum (FCS), 0.1 mg/mL streptomycin and 100 U/mL penicillin. Supernatant was harvested every 2–3 days for 2 weeks. Supernatants were filtered (0.22 µM) before purification. IgG, Fab fragments, or scFvs were captured on protein L columns (HiTrap, GE Healthcare) or CH1 capture select columns (Thermo Fisher Scientific), eluted with 0.1 M glycine-HCl pH 3 and neutralized with 1 M Tris-HCl pH 8. Alternatively, scFvs were purified by IMAC (HiTrap, GE Healthcare) and eluted with 50 mM Tris-HCl, 0.5 M NaCl 0.5 M imidazol pH 7.4. Proteins were size excluded on a Superdex 200 column using PBS supplemented with 150 mM NaCl (GE Healthcare).

## ELISA

EIA/RIA plates were coated with 10 µg/mL NeutrAvidin in PBS (100 µL/well, Thermo Scientific) and incubated ON/4 °C. Plates were blocked with 5% skim milk powder in PBST (300 µL/well) for 1 h/RT with gentle agitation. Equal amounts of biotinylated pHLA variants (normalized to 300 ng/mL) were captured for 1 h/RT, followed by addition of 0.5 µg/mL purified TCR-like antibodies, 5 µg/mL purified Fab fragments or purified scFv, or unknown concentrations of soluble scFv or phage during single-clone screening. Bound scFv were detected with mouse anti-Myc tag antibody (Invitrogen, 1:5,000) and anti-mouse-HRP (GE Healthcare, 1:2,000). Bound hIgG1 were detected with anti-hIgG-ALP (Sigma Aldrich, 1:3,000), Fab fragments with anti-hCκ-ALP (Sigma, 1:3,000), and phage particles with anti-M13-HRP (Amersham Biosciences, 1:5,000). For estimation of scFv expression levels in R3 outputs, 10 µl of each medium and periplasmic fractions were combined and diluted to 100 µl using PBS and directly coated onto EIA/RIA plates. scFvs were detected using HRP-conjugated protein L (GenScript, 1:1000). All antibodies and protein L were diluted in PBST. Plates were developed with TMB solution (Calbiochem) and read at 450 nm using a microplate reader (Tecan sunrise) after stopping the enzymatic reaction by addition of 1 M HCl. Alternatively, the plates were developed with 1 mg/mL phosphatase substrate (Sigma Aldrich) in diethanolamine buffer and read at 405 nm.

## Thermostability analysis by nanoDSF

Purified Fab fragments were diluted to 0.2 mg/mL in PBS and 10 µL were transferred to glass capillaries (NanoTemper) in triplicates. Samples were subjected to a temperature from 20 °C to 95 °C, with 1 °C/min increments using a Prometheus nanoDSF (NanoTemper). An excitation wavelength of 295 nm was used and emission was measured at 330 nm and 350 nm. Data was collected and analyzed using AB-Protein PR.ThermControl V2.12 to determine Fab melting temperatures.

## Binding analysis by SPR

SPR was conducted using a Biacore T200 (GE Healthcare) as previously described *(8)*. Briefly, NeutrAvidin (10 µg/mL in 10 mM sodium acetate, pH 4.5) was coupled onto a CM3 sensor chip to 1000 response units (RU) by amine coupling before capture of 80-90 RU of soluble, recombinant, biotinylated pHLA (1 µg/mL). Kinetics and affinity of Fab fragments was determined using either single cycle kinetics or a multi cycle method. For off-rate ranking, all samples were used at 0.5 μM. All samples were diluted in PBS supplemented with 0.05% (v/v) surfactant P20 and run at 30 µL/min at 25°C. Data was fitted to a 1:1 Langmuir binding model after buffer subtraction and NeutrAvidin-reference-cell subtraction using the T200 Evaluation Software v1.0.

## Flow cytometric analyses of stained A 20 B cells

Murine A20 B cell lymphoma cells had previously been engineered to express HLA-DQ2.5 with different peptides covalently linked to the HLA β-chain *(2, 16)*. Cells were stained using 5 µg/mL hIgG1 mAbs together with rat anti-mouse CD16/CD32 block (1:200; BD Biosciences). Bound mAbs were detected using biotinylated goat F(ab′)2 anti-hIgG (2 µg/mL; Southern Biotech) followed by streptavidin R-PE (2 µg/mL; Invitrogen). To control for pHLA expression, A20 cells were stained with biotinylated 2.12.E11 mIgG1 (5 µg/mL) followed by streptavidin R-PE as before. An equal number of cells (at least 100,000) were stained on ice using V-bottom 96-well plates. PBS supplemented with 2% FCS was as staining buffer. Data were acquired using an Attune NxT Flow Cytometer (Thermo Fisher Scientific).

# Supplementary Figures


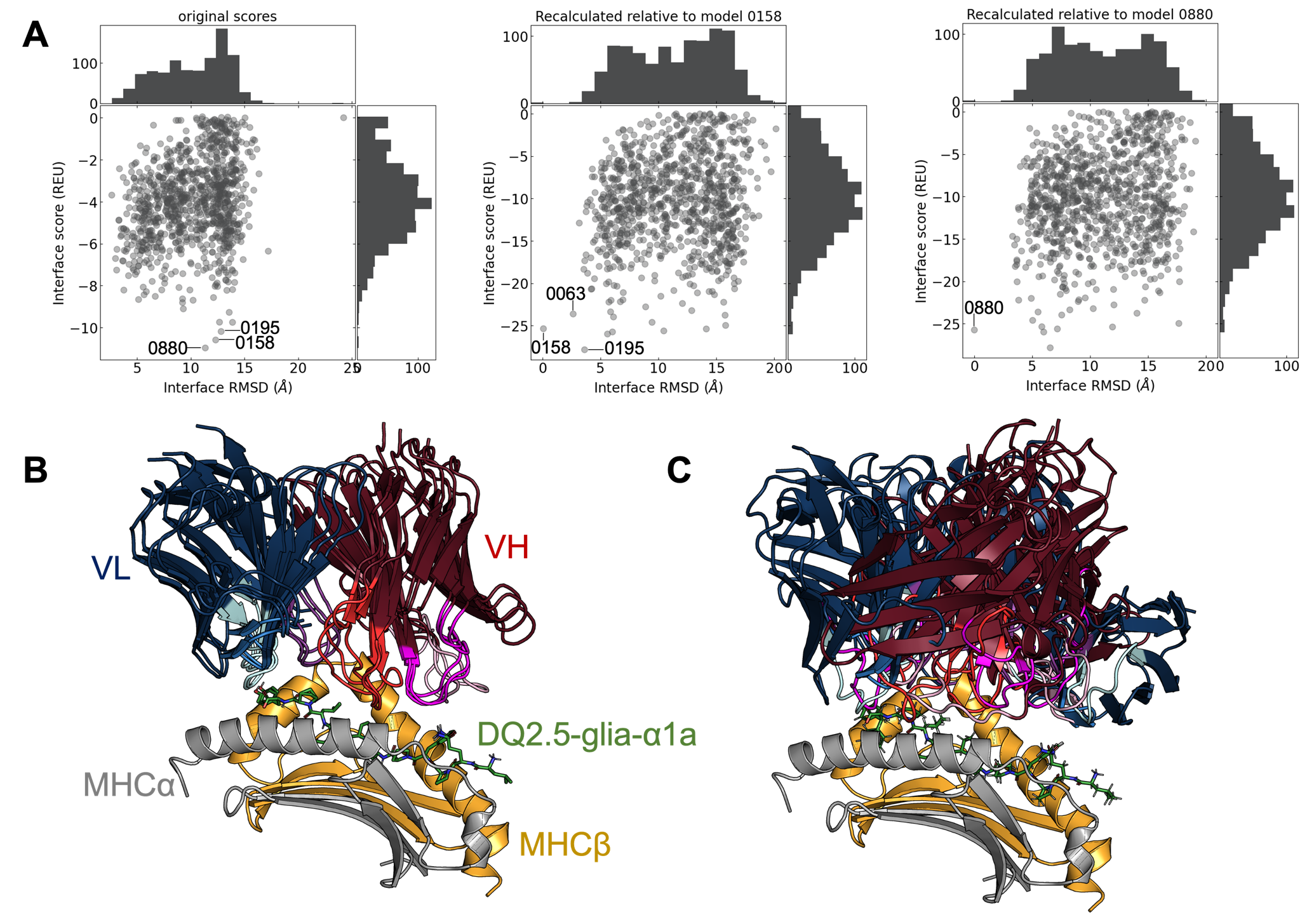


**Figure S1. Model selection.** **A)** 1,000 models of the antibody in complex with pHLA were generated using Rosetta SnugDock and their interface scores are plotted against the interface RMSD relative to the input orientation (“original scores”). Low scoring models are annotated. In order to identify potential “energy funnels”, where a number of structurally similar models has a lower Rosetta energy than the bulk of the models, the interface scores and RMSDs were recalculated to the two low scoring models 0158 and 0880 after refinement as described in *(1)*. Low scoring low-RMSD models are annotated. **B)** Three highly similar low scoring models were identified after recalculating interface RMSDs relative to 0158. The three models are overlaid, and chains are annotated. These models were selected for analysis. **C)** For comparison 5 random models are overlaid with chains colored as in B.


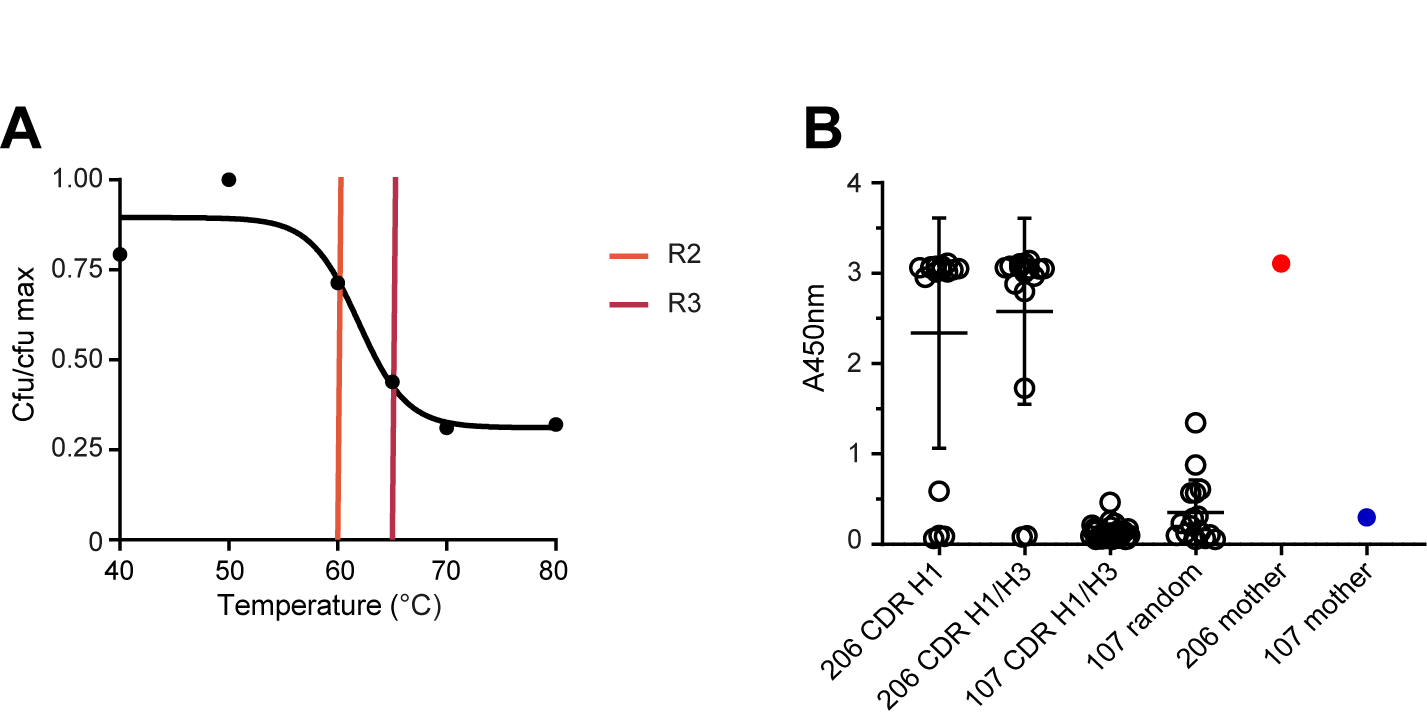


**Figure S2. Thermostability of mAb 107 and scFv expression levels. A)** Phage samples displaying the first-generation antibody 107 at HV on coat protein pIX were heated at different temperatures for 10 min, followed placement on ice. Residual binding to protein L was determined by incubating samples with protein L-coated ELISA plates, followed by trypsin elution and infectious titration of recovered particles. The melting temperature (Tm) of mAb 107 was estimated to 62 °C by fitting a sigmoid function to the data. Heat challenge temperatures used for R2 and R3 of selection in the thermo branch are indicated (red and orange lines). **B)** Single clones derived from the R3 selection outputs from the current selection (107) and a previous selection (206, *(8)*) were expressed as scFv along with the respective mother clones. A mix of supernatants and periplasmic fractions were assessed for binding to protein L to determine expression levels of correctly folded scFv. Each dot represents one clone. The following number of clones were screened from each output; 206 CDR H1 (n = 16), 206 CDRH1/H3 (n = 16), 107 CDR H1/H3 (n = 32), 107 random (n = 16). Error bars illustrate mean ± SD.


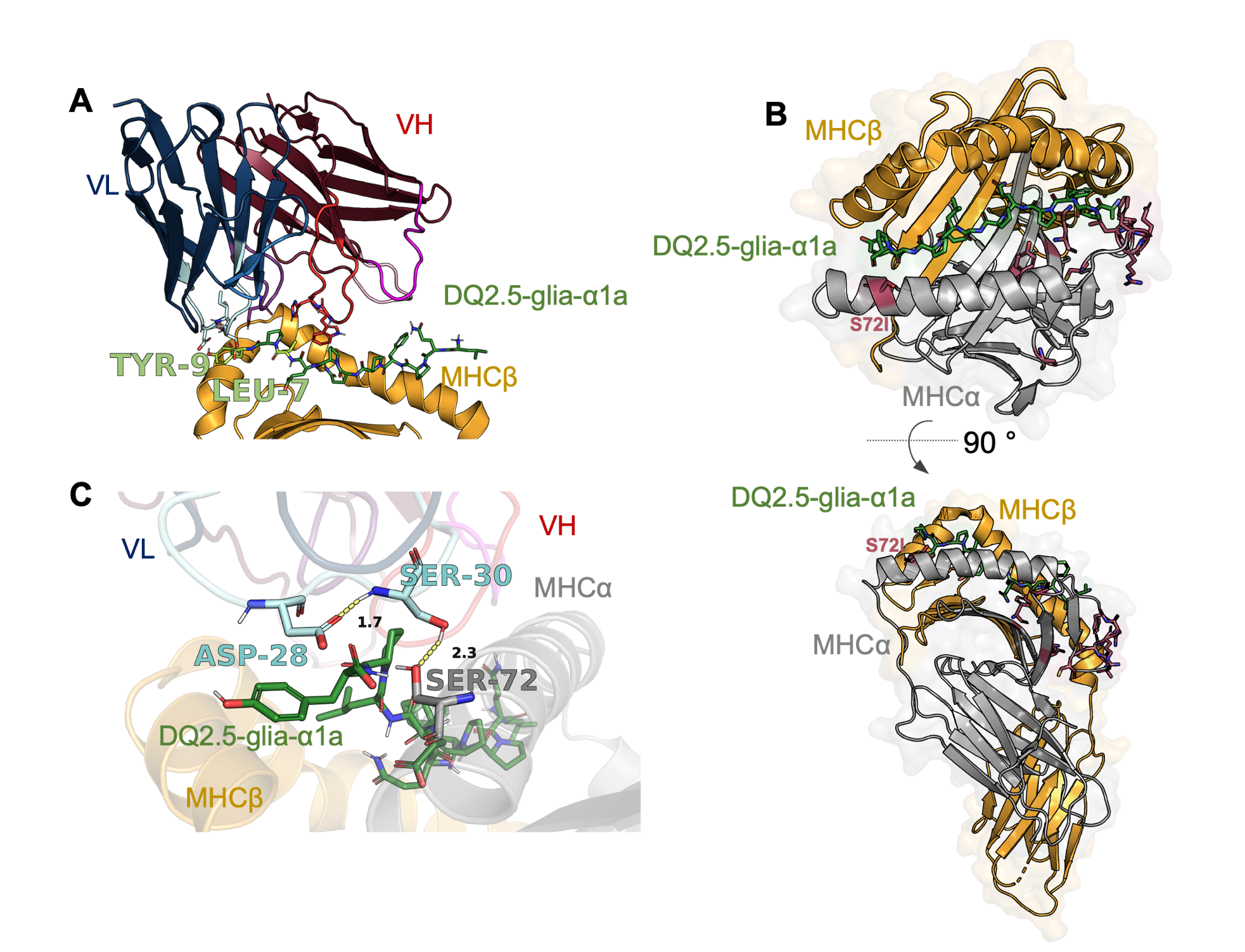


**Figure S3. Structural hypotheses for experimentally observed fine specificty.** **A)** The antibodies discriminate between the highly similar epitopes of HLA-DQ2.5:DQ2.5-glia-α1 and HLA-DQ2.5:DQ2.5-ω1. These epitopes differ at positions 7 and 9 of the peptide (highlighted in light green). The structural model of 107 in complex with pHLA is shown as a cartoon. The peptide and antibody residues within 4 Å of positions p7 and p9 are shown in stick representation. **B)** Crystal structure of HLA-DQ2.5:DQ2.5-glia-α1a (PDB 4OZI) *(6)* shown as cartoon and transparent surface. HLA-DQ2.5/HLA-DQ2.2 polymorphisms are shown in stick representation and highlighted in red. The exposed S72I polymorphism is annotated. **C)** The antibodies also discriminate between the target HLA (HLA-DQ2.5) and the closely related variant HLA-DQ2.2. Only two polymorphisms in the α-chain are potentially visible to a TCR or antibody, and out of these position Ser72 is predicted to form a direct polar interaction with residue Ser30 of the antibody light chain, offering a possible explanation for the observed specificity. Chains are color-coded and annotated as in A.

**Supplementary Table 1. Design strategy for targeted libraries.**

| Library name | Randomization | Primary transformants | Selection  branch |
| --- | --- | --- | --- |
| CDR H1^1^ | CDR H1 randomization  length increased by 1 or 2 residues | 3.6E9 | Competition |
| CDR H3^1^ | CDR H3 randomization  fully random or retained W100 | 3.2E9 | Competition |
| CDR H1/H3^2^ | CDR H1 + CDR H3 | 6.8E9 | Thermal |

^1^ The CDR H1 and CDR H3 libraries based on mAb 107 were designed to introduce new or generate improved interactions between the respective mAb heavy chain CDR loops and the target pHLA. Degenerate NNK oligonucleotides were used.

^2^ The CDR H1/H3 library contained a blend of CDR H1 and CDR H3 loop mutants and was selected in the thermal branch only.

| **Supplementary Table 2. Sequence alignment of clones** | | | | | | | |
| --- | --- | --- | --- | --- | --- | --- | --- |
| **Clone** | **FR1** | **CDR1** | **FR2** | **CDR2** | **FR3** | **CDR3** | **FR4** |
| **VH** | 1 25 | 26 33 | 34 50 | 51 57 | 58 92 | 93 102 | 103 113 |
| 107 | QVQLQQSGPGLVKPSQTLSLTCAIS | GDSVSSNSAA | WNWIRQSPSRGLEWLGR | TYYRSKWYN | DYAVSVKSRITINPDTSKNQFSLQLNSVTPEDTAVYYC | ARDSSSGWHPYGMDV | WGQGTTVTVSS |
| 4.5D | ......................... | .......... | ................. | ......... | ...................................... | ....TT..NA..... | ........... |
| 4.6D | ......................... | .......... | ................. | ......... | ...................................... | ....T.......... | ........... |
| 4.6C | ......................... | .......... | ................. | ......... | ...................................... | ....TT..GA..... | ........... |
| 4.7C | ......................... | .......... | ................. | ......... | ...................................... | ...RTT......... | ........... |
| 5.6A | ......................... | ......S... | ................. | ......... | ...................................... | ............... | ........... |
| 15.6A | .........E............... | .......... | ................. | ......... | ...................................... | ............... | ........... |
| **VL^a^** | 1 26 | 27 32 | 33 49 | 50 52 | 53 88 | 89 97 | 98 107 |
| 107 | DIQVTQSPSFLSASVGDRVTITCRAS | HDISSY | LAWYQHKPWKAPKLLIH | AAS | ILQSGVPSRFSGSGSGTEFTLTISSLQPEDFATYYC | QDLNSYPL | FGQGTRLEIK |
| 5.6A | .V........................ | ...... | ................. | ... | V................................... | .N...... | .......... |
| 15.6A | ..R.....................T. | ...... | ................. | ... | .................................... | ........ | .......... |

**^a^** 4.5D, 4.6D, 4.6C, 4.7C share VL sequence with 107

Amino acid changes in selected clones are shown and position highlighted in grey. Numbering according to the Chothia numbering scheme. CDR loops defined by the IMGT database.

| **Supplementary Table 3. Kinetics and affinity of affinity matured variants.** | | | | | | |  |
| --- | --- | --- | --- | --- | --- | --- | --- |
|  | Kinetics and affinity | | | | Steady state^c^ | |  |
| Clone | k_on_ (M^-1^s^-1^) | k_off_ (s^-1^) | K_D_ (M) | SE K_D_ (M) | K_D_ (M) | SE K_D_ (M) |  |
| **HLA-DQ2.5:DQ2.5-glia-α1a binders** | | | | | | |  |
| scFv 107^a,b^ | 2.89x10^5^ | 0.02151 | 7.43x10^-8^ | 7.44x10^-8^ | 6.70x10^-8^ | 1.30x10^-8^ |  |
| Fab 107^a^ | 2.24x10^5^ | 0.01601 | 7.14x10^-8^ | 5.54x10^-8^ | 7.41x10^-8^ | 1.20x10^-9^ |  |
| Fab 107^d^ | 2.377x10^5^ | 0.01698 | 7.145x10^-8^ |  | NA | NA |  |
| Fab 4.7C^d^ | 3.776x10^5^ | 9.819x10^-5^ | 2.600x10^-10^ |  | NA | NA |  |
| Fab 4.5D^d^ | 3.830x10^5^ | 1.657x10^-3^ | 4.327x10^-9^ |  | NA | NA |  |
| Fab 4.6C^d^ | 6.972x10^5^ | 2.617x10^-4^ | 3.754x10^-10^ |  | NA | NA |  |
| Fab 4.6D^d^ | 4.638x10^5^ | 5.413x10^-4^ | 1.167x10^-9^ |  | NA | NA |  |
| Fab 5.6A^d^ | 1.002x10^6^ | 3.442x10^-3^ | 3.436x10^-9^ |  | NA | NA |  |
| Fab 15.6A^d^ | 5.883x10^5^ | 2.209x10^-3^ | 3.755x10^-9^ |  | NA | NA |  |
| Fab 4.7C^e^ | 9.007x10^5^ | 1.770x10^-4^ | 1.966x10^-10^ | 5.40x10^-7^ | NA | NA |  |
| Fab 4.7C^e^ | 6.697x10^5^ | 9.685x10^-5^ | 1.446x10^-10^ | 5.60x10^-7^ | NA | NA |  |
| Fab 4.7Cplus^e^ | 1.829x10^6^ | 5.861x10^-5^ | 3.204x10^-11^ | 3.80x10^-7^ | NA | NA |  |
| Fab 4.7Cplus^e^ | 2.048x10^6^ | 1.550x10^-5^ | 7.569x10^-12^ | 5.60x10^-7^ | NA | NA |  |
| Kinetics were determined by fitting data to a 1:1 Langmuir binding model. | | | | | | | |
| ^a^ Determined from single cycle kinetics. | | | | | | | |
| ^b^ Data for scFv 107 reported in *(2)*.  ^c^ Steady state K_D_ was derived from the single cycle kinetics runs.  ^d^ Determined from one concentration of protein in off-rate screening. K_D_s obtained with this method disregarded when calculating average values.  ^e^ Values derived from multicycle runs.  NA = Not available | | | | | | | |

# Supplementary References

1. B. D. Weitzner, J. R. Jeliazkov, S. Lyskov, N. Marze, D. Kuroda, R. Frick, J. Adolf-Bryfogle, N. Biswas, R. L. Dunbrack, J. J. J. Gray, Modeling and docking of antibody structures with Rosetta, *Nat. Protoc.* **12**, 401–416 (2017).

2. L. S. Høydahl, L. Richter, R. Frick, O. Snir, K. S. Gunnarsen, O. J. B. Landsverk, R. Iversen, J. R. Jeliazkov, J. J. Gray, E. Bergseng, S. Foss, S.-W. Qiao, K. E. A. Lundin, J. Jahnsen, F. L. Jahnsen, I. Sandlie, L. M. Sollid, G. Å. Løset, Plasma Cells Are the Most Abundant Gluten Peptide MHC-expressing Cells in Inflamed Intestinal Tissues From Patients With Celiac Disease., *Gastroenterology* **156**, 1428-1439.e10 (2019).

3. C.-Y. Kim, H. Quarsten, E. Bergseng, C. Khosla, L. M. Sollid, Structural basis for HLA-DQ2-mediated presentation of gluten epitopes in celiac disease., *Proc. Natl. Acad. Sci. U. S. A.* **101**, 4175–4179 (2004).

4. A. Sircar, J. J. J. J. J. J. Gray, SnugDock: Paratope Structural Optimization during Antibody-Antigen Docking Compensates for Errors in Antibody Homology Models, *PLoS Comput. Biol.* **6**, e1000644 (2010).

5. J. R. Jeliazkov, R. Frick, J. Zhou, J. J. Gray, Robustification of RosettaAntibody and Rosetta SnugDock, *PLoS One* **16**, 1–20 (2021).

6. J. Petersen, V. Montserrat, J. R. Mujico, K. L. Loh, D. X. Beringer, M. van Lummel, A. Thompson, M. L. Mearin, J. Schweizer, Y. Kooy-Winkelaar, J. van Bergen, J. W. Drijfhout, W.-T. Kan, N. L. La Gruta, R. P. Anderson, H. H. Reid, F. Koning, J. Rossjohn, T-cell receptor recognition of HLA-DQ2–gliadin complexes associated with celiac disease, *Nat. Struct. Mol. Biol.* **21**, 480–488 (2014).

7. G. Å. Løset, N. Roos, B. Bogen, I. Sandlie, Expanding the versatility of phage display II: Improved affinity selection of folded domains on protein VII and IX of the filamentous phage, *PLoS One* **6** (2011), doi:10.1371/journal.pone.0017433.

8. R. Frick, L. S. Høydahl, J. Petersen, M. F. du Pré, S. Kumari, G. Berntsen, A. E. Dewan, J. R. Jeliazkov, K. S. Gunnarsen, T. Frigstad, E. S. Vik, C. Llerena, K. E. A. Lundin, S. Yaqub, J. Jahnsen, J. J. Gray, J. Rossjohn, L. M. Sollid, I. Sandlie, G. Å. Løset, A high-affinity human TCR-like antibody detects celiac disease gluten peptide-MHC complexes and inhibits T cell activation., *Sci. Immunol.* **6** (2021), doi:10.1126/sciimmunol.abg4925.

9. R. Tonikian, Y. Zhang, C. Boone, S. S. Sidhu, Identifying specificity profiles for peptide recognition modules from phage-displayed peptide libraries., *Nat. Protoc.* **2**, 1368–1386 (2007).

10. G. Å. Løset, B. Bogen, I. Sandlie, Expanding the versatility of phage display I: Efficient display of peptide-tags on protein VII of the filamentous phage, *PLoS One* **6**, 1–7 (2011).

11. L. S. Høydahl, N. R. Nilssen, K. S. Gunnarsen, M. F. du Pré, R. Iversen, N. Roos, X. Chen, T. E. Michaelsen, L. M. Sollid, I. Sandlie, G. Å. Løset, Multivalent pIX phage display selects for distinct and improved antibody properties, *Sci. Rep.* **6**, 39066 (2016).

12. N. R. Nilssen, T. Frigstad, S. Pollmann, N. Roos, B. Bogen, I. Sandlie, G. Å. Løset, DeltaPhage--a novel helper phage for high-valence pIX phagemid display., *Nucleic Acids Res.* **40**, e120 (2012).

13. L.-E. Fallang, S. Roh, A. Holm, E. Bergseng, T. Yoon, B. Fleckenstein, A. Bandyopadhyay, E. D. Mellins, L. M. Sollid, Complexes of two cohorts of CLIP peptides and HLA-DQ2 of the autoimmune DR3-DQ2 haplotype are poor substrates for HLA-DM., *J. Immunol.* **181**, 5451–5461 (2008).

14. K. S. Gunnarsen, E. Lunde, P. E. Kristiansen, B. Bogen, I. Sandlie, G. a Løset, Periplasmic expression of soluble single chain T cell receptors is rescued by the chaperone FkpA., *BMC Biotechnol.* **10**, 8 (2010).

15. G. Berntzen, E. Lunde, M. Flobakk, J. T. Andersen, V. Lauvrak, I. Sandlie, Prolonged and increased expression of soluble Fc receptors, IgG and a TCR-Ig fusion protein by transiently transfected adherent 293E cells., *J. Immunol. Methods* **298**, 93–104 (2005).

16. K. S. Gunnarsen, L. S. Høydahl, L. F. Risnes, S. Dahal-Koirala, R. S. Neumann, E. Bergseng, T. Frigstad, R. Frick, M. F. du Pré, B. Dalhus, K. E. Lundin, S.-W. Qiao, L. M. Sollid, I. Sandlie, G. Å. Løset, A TCRα framework-centered codon shapes a biased T cell repertoire through direct MHC and CDR3β interactions., *JCI insight* **2** (2017), doi:10.1172/jci.insight.95193.
